# Two-step synthesis of fatty acid ethyl ester from soybean oil catalyzed by *Yarrowia lipolytica *lipase

**DOI:** 10.1186/1754-6834-4-6

**Published:** 2011-03-02

**Authors:** Yonghong Meng, Guili Wang, Na Yang, Zhiqi Zhou, Yuejuan Li, Xiaomei Liang, Jinnan Chen, Ying Li, Jilun Li

**Affiliations:** 1 State Key Laboratories for Agrobiotechnology and College of Biological Sciences, China Agricultural University, Beijing 100193, People's Republic of China; 2 New Bio-Energy Engineering and Technology Research Center, Qinhuangdao Leading Science & Technology Development Co., Ltd, Hebei 066004, People's Republic of China

## Abstract

**Background:**

Enzymatic biodiesel production by transesterification in solvent media has been investigated intensively, but glycerol, as a by-product, could block the immobilized enzyme and excess *n*-hexane, as a solution aid, would reduce the productivity of the enzyme. Esterification, a solvent-free and no-glycerol-release system for biodiesel production, has been developed, and two-step catalysis of soybean oil, hydrolysis followed by esterification, with *Yarrowia lipolytica *lipase is reported in this paper.

**Results:**

First, soybean oil was hydrolyzed at 40°C by 100 U of lipase broth per 1 g of oil with approximately 30% to 60% (vol/vol) water. The free fatty acid (FFA) distilled from this hydrolysis mixture was used for the esterification of FFA to fatty acid ethyl ester by immobilized lipase. A mixture of 2.82 g of FFA and equimolar ethanol (addition in three steps) were shaken at 30°C with 18 U of lipase per 1 gram of FFA. The degree of esterification reached 85% after 3 hours. The lipase membranes were taken out, dehydrated and subjected to fresh esterification so that over 82% of esterification was maintained, even though the esterification was repeated every 3 hours for 25 batches.

**Conclusion:**

The two-step enzymatic process without glycerol released and solvent-free demonstrated higher efficiency and safety than enzymatic transesterification, which seems very promising for lipase-catalyzed, large-scale production of biodiesel, especially from high acid value waste oil.

## Background

Biodiesel fuel (fatty acid methyl ester or ethyl ester) is renewable and biodegradable and has "environmentally friendly" features; for example, it can be produced from animal and vegetable oils, and the number of carbon atoms present in the exhaust is equal to that initially fixed from the atmosphere [1]. The number of researchers studying biodiesel has steadily increased during the past decade, and methods of large-scale biodiesel production based on acid or alkaline catalysis have been widely used. However, they have many well-known drawbacks, including the difficulty of recycling glycerol, the need to eliminate catalyst and salt and their energy-intensive nature.

Designed to overcome these drawbacks, enzymatic methods of producing fatty acid methyl ester (FAME) or fatty acid ethyl ester (FAEE) from soybean oil and alcohol are afforded and dominated by transesterification reaction. The advantages of transesterification are that the enzyme can be reused and that the operating temperature is lower (40°C) than that in other techniques. Its disadvantages are that the catalysis activity of lipase is inhibited by alcohol, the immobilized enzyme is blocked by a by-product of glycerol and the production intensity of lipase is decreased by organic solvents such as *n*-hexane [2] and *tert*-butyl alcohol [3,4].

Safer and more environmentally friendly enzymatic methods can be developed on the basis of solvent- and glycerol-free catalysis. When the catalytic process does not involve an organic solvent, lower cost, higher substrate concentration and greater production volume can be achieved. For example, Du *et al. *[5] studied Novozyme 435-catalyzed transesterification of soybean oil and methyl acetate directly for biodiesel production, and a yield of 92% was obtained. Moreover, Watanabe *et al. *[6,7] transferred acid oil to FAME without use of an organic solvent by a two-step conversion process involving hydrolysis of acylglycerol by *C. rugosa *lipase followed by esterification of free fatty acid (FFA) to FAME by immobilized *C. antarctica *lipase. Inhibition of glycerol on immobilized lipase was eliminated during hydrolysis, and a high degree (98%) of esterification, 40 cycles reusing immobilized lipase, was achieved. In the studies by Watanabe *et al.*, esterification was carried out in two steps once more. The first esterification step was performed at 30°C with 1.0% weight lipase in the reaction mixture. The second esterification step was performed in the presence of absorbing solvent under conditions similar to those in the first step, following dehydration of the first esterification product.

Previous studies have documented the reaction condition and stability of immobilized lipase in solvent-free catalytic systems, but the level of production has not been fully considered, such as the high costs of immobilization of lipase and the long time required for esterification (each batch reaction took 24 hours). Here we describe a promising two-step enzymatic process for the conversion of soybean oil to FAEE: (1) Soybean oil is hydrolyzed to fatty acid catalyzed by *Yarrowia lipolytica *lipase crude broth, and FFA is distilled from hydrated soybean oil mixture; and (2) FAEE is esterified from FFA and ethanol by the immobilized *Y. lipolytica *lipase on fabric membranes. The entire esterification process without organic solvent takes only 3 hours, uses only one kind of comparatively cheap lipase and the inhibition of glycerol disappears.

## Results

### Hydrolysis reaction

The efficiency of hydrolysis in lipase-catalyzed systems is affected by many factors, including lipase, the ratio of oil to water and the degree of mixing of oil and water. To find optimal conditions for hydrolysis of acylglycerols using sodium stearate as an emulsifier to promote mixing, we studied the effects of lipase concentration, the amount of water in the reaction solution and the course of the hydrolysis reaction.

#### Effect of emulsifier

**Figure 1:**
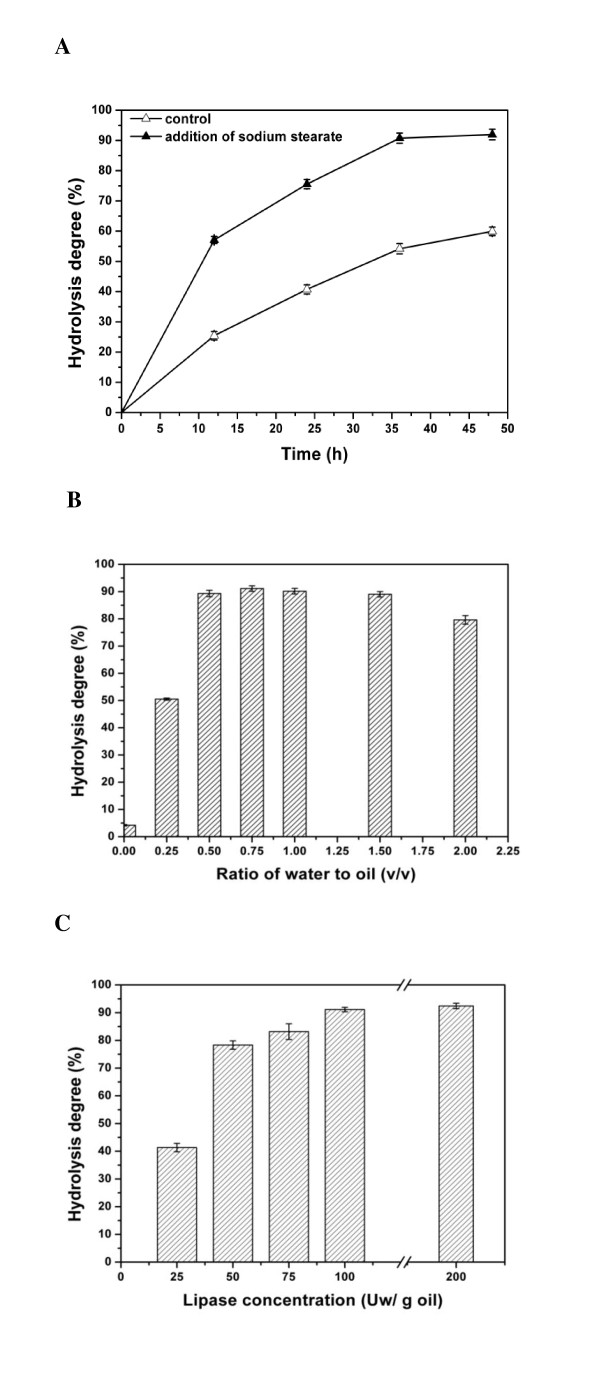
**Effect of emulsifier, water content and lipase concentration on hydrolysis reaction**. **(A) **Effect of emulsifier on hydrolysis reaction. The reaction system comprised 2 g of soybean oil, 200 Uw lipase broth, 1.2 g of water and 5% (wt/vol) added sodium stearate at 40°C and spun at 130 rpm. No emulsifier was added for the control reaction. **(B) **Effect of water content on hydrolysis reaction. The reaction system comprised 2 g of soybean oil, 200 Uw lipase broth, various quantities of water to produce water, oil ratios as indicated and 0.1 g of sodium stearate at 40°C for 36 hours and spun at 130 rpm. **(C) **Effects of lipase concentration on hydrolysis reaction. The reaction system comprised 2 g of soybean oil, various quantities of lipase broth to produce concentrations as indicated, 1.2 g of water and 0.1 g of sodium stearate at 40°C for 36 hours and spun at 130 rpm.

Sodium stearate is an emulsifier that promotes the mixing of oil and water. Since sodium stearate can be converted to stearic acid by the addition of acid at the end of the reaction, it hardly affects the results of hydrolysis. Various quantities of sodium stearate solution were mixed with soybean oil, and the reaction was started by adding lipase broth. The degree of hydrolysis after 36 hours was >90% with 5% (wt/vol) sodium stearate addition, in contrast to 60% without sodium stearate addition (Figure 1A), and oil and aqueous phases mixed well.

#### Effect of water content

Excess water slows the velocity of hydrolysis because it reduces the concentration of lipase. The maximal efficiency of reactions was observed for ratios 0.25 to 1.5 (volume ratio) of water to oil. Larger ratios decreased the hydrolysis reaction and the efficiency of lipase (Figure 1B).

#### Effect of lipase concentration

Hydrolysis degree was determined for lipase concentrations ranging from 25 to 400 Uw per 1 g of soybean oil. For 25 Uw/g lipase, the hydrolysis degree was low. As the lipase concentration was increased, the degree of hydrolysis also increased, reaching a maximal value >90% for the enzyme concentration 100 Uw/g (Figure 1C). Further increases in enzyme concentration did not increase the degree of hydrolysis.

#### Course of hydrolysis reaction

**Figure 2:**
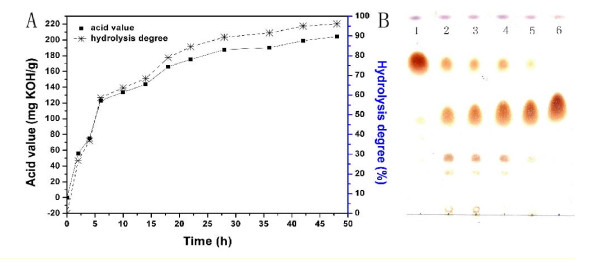
**Time course of hydrolysis reaction**. **(A) **Time course of acid value and hydrolysis degree. Chromatography at various time points (time point 1, 0 hour; time point 2, 6 hours; time point 3, 12 hours; time point 4, 24 hours; time point 5, 36 hours; time point 6, 48 hours). **(B) **Time course of composition of oil phase during hydrolysis reaction analyzed as described in Methods. Thin layer chromatography analysis of (top to bottom) triglycerides, fatty acids, diglycerides and glycerol monoesters. The reaction system comprised 200 g of soybean oil, 20,000 Uw lipase broth, 120 g of water and 10 g of sodium stearate at 40°C for 36 hours and spun at 180 rpm.

For large-scale hydrolysis, lipase was added (concentration 100 Uw/g oil) to a system containing 200 g of soybean oil, 20,000 Uw lipase broth, 120 ml of water and 10 g of sodium stearate. Acid value and hydrolysis degree are shown in Figure 2A. At prophase, the hydrolysis velocity was high and the hydrolysis degree reached 65% after 10 hours, 90% after 36 hours, and 92.5% at 48 hours, respectively. Total acid value at 48 hours was 185 mg of KOH per 1 g of oil. The composition of triglycerides, diglycerides, glycerol monoesters and fatty acids was analyzed by thin layer chromatography (TLC) (Figure 2B).

Following the hydrolysis reaction, distillates were collected at 220°C to 260°C as described in Methods. Gas chromatography (GC) revealed the following fatty acid composition: 20.03% palmitic acid, 4.85% stearic acid, 24.17% oleic acid, 47.03% linoleic acid and 3.92% linolenic acid.

### Esterification reaction

Before catalyzed esterification, the viscosity of the reaction mixture was analyzed. The fatty acid or mixture of 2.82 g of fatty acid and 587 μl of ethanol had the lowest viscosity (8.75 cP and 8.75 cP, respectively) compared to soybean oil and mixture of 2.82 g of soybean oil and 587 μl of ethanol (46.5 cP and 18.75 cP, respectively). These viscosity data may show that fatty acid and ethanol were commixed well and can be esterified by lipase catalysis in the absence of organic solvent as Watanabe *et al. *reported [7]. So, fatty acids distilled from hydrolysis mixture, characterized by approximately 147 to 153 ppm water content, were prepared for esterification. The amount of immobilized lipase, the ratio of ethanol to fatty acid, the manner of ethanol addition and water content were all examined in a lipase-catalyzed esterification reaction to find the optimal conditions for the reaction.

#### Effect of amount of immobilized lipase

**Figure 3:**
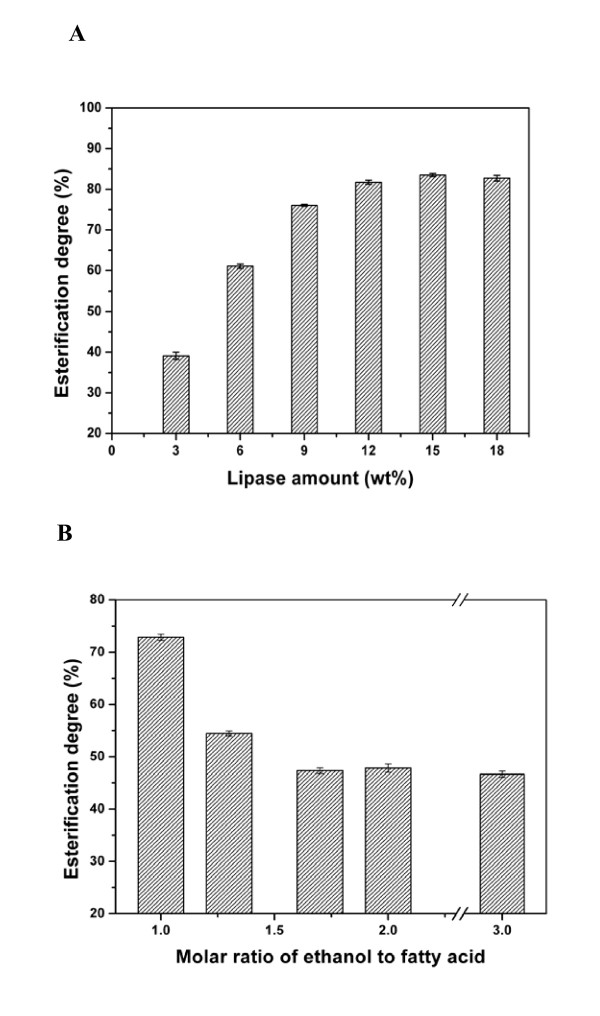
**Effects of immobilized lipase amount and ratio of ethanol to fatty acid on esterification reaction**. **(A) **Effect of immobilized lipase amount on esterification reaction. The reaction system comprised 2.82 g of fatty acid, 580 μl of ethanol added at one time and the addition of various amounts of immobilized lipase added at 30°C for 3 hours and spun at 190 rpm. **(B) **The effect of the ratio of ethanol to fatty acid on the esterification reaction. The reaction system comprised 2.82 g of fatty acid, various amounts of ethanol added at one time and 0.33 g of immobilized lipase at 30°C for 3 hours and spun at 190 rpm.

The esterification activity of immobilized lipase, prepared as described in the Lipase activity determination section, was 150 Ue per 1 g of membrane. The amount of immobilized lipase was determined as the weight percentage of lipase to fatty acid. As lipase content increased from 0% to 18% (wt/vol), the esterification degree increased gradually (Figure 3A). The increase in lipase content beyond 12% did not result in a significant increase in esterification degree during a 3-hour reaction, indicating that this value is appropriate for catalysis in this system.

#### Effect of ratio of ethanol to fatty acid

The addition of excessive alcohol to the reaction mixture is often used to enhance reaction velocity and esterification degree. However, in this study, maximal esterification was obtained with an ethanol:fatty acid ratio of 1:1, and higher ratios produced a lower degree of esterification (Figure 3B). This finding suggests that excessive ethanol suppresses lipase activity.

#### Effect of manner of ethanol addition

Ethanol serves as a reaction substrate; however, at high concentrations, it also denatures proteins, including enzymes. We tried adding ethanol in a series of steps to minimize its denaturing effect. A 2:1 molar ratio of ethanol to fatty acid was achieved by 1-step, 3-step, 5-step and 10-step addition methods. The esterification reaction was inhibited when ethanol was added by the one-step method, and the esterification degree was about 50%. Moreover, the esterification degree did not increase with the extension of the reaction time. However, the esterification degree increased to 81.6% as larger numbers of steps were used (Table 1).

**Table 1:** Effect of manner of ethanol addition on esterification^a^

			Mean esterification ± SD, %		
				
Addition method	Ethanol:fatty acid molar ratio	Time, hours	Treatment	Control	Rate of increase, %
Three-step	2:1	3	60 ± 0.643	49.8 ± 0.493	20.5
Five-step	2:1	5	76.7 ± 0.794	51.5 ± 0.5	48.9
Ten-step	2:1	24	81.6 ± 0.6	54.1 ± 1.49	50.8

^a^Control reaction used one-step addition of ethanol. Reaction system comprised 2.82 g of fatty acid, 580 × 2 μl of ethanol added in various ways, 0.33 g of immobilized lipase at 30°C and spun at 190 rpm. SD, standard deviation.

#### Effect of water

Water has important dual roles in esterification systems: (1) It is essential to maintaining lipase conformation and catalytic activity, and (2) it is the product of esterification and affects the equilibrium state of the esterification or hydrolysis reactions. So, the effects of the addition or removal of water were investigated in the esterification system.

**Figure 4:**
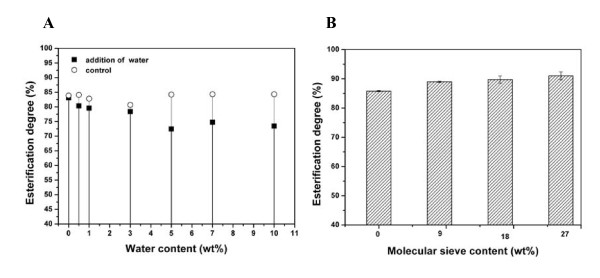
**The effect of water on the esterification reaction**. **(A) **The effect of adding water into the reaction mixture. Solid squares represent the esterification degree with various amounts of added water. Open circles indicate the esterification degree of the fresh esterification reaction catalyzed by the dried immobilized lipase membrane. **(B) **The effect of the addition of various amounts of molecular sieves. The reaction system comprised 2.82 g of fatty acid, 580 μl of ethanol added in various ways and 0.33 g of immobilized lipase at 30°C for 5 hours and spun at 190 rpm.

Figure 4 shows a direct comparison of the yield of FAEE with the addition of various water concentrations from approximately 0% to 10% (vol/vol). The results showed that water content of < 0.5% had almost no effect on esterification. On the other hand, the efficiency of esterification was low for water content >1% (Figure 4A). However, if the immobilized lipase membrane was taken out, dried at 40°C and reused in fresh esterification, its catalytic activity resumed (Figure 4A). This finding suggests that excess water (>0.5%) inhibits esterification by affecting the reaction equilibrium and that the inhibition is abolished by removing the water released in the esterification process.

Molecular sieves were added in the esterification system to absorb and remove released water. Molecular sieves with content ranging from 0% to 27% (wt/vol) were added experimentally to the system. The addition of 9% molecular sieves resulted in an increase in the degree of esterification from 85% to 90% (Figure 4B).

#### Course of esterification reaction

**Figure 5:**
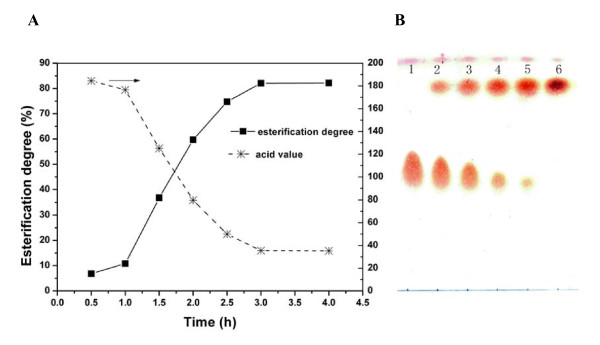
**Time course of the esterification reaction**. **(A) **The time course of esterification degree and acid value. Chromatography performed at various time points (time point 1, 0 hour; time point 2, 1 hour; time point 3, 1.5 hours; time point 4, 2 hours; time point 5, 3 hours; time point 6, 12 hours). **(B) **Time course of the composition of oil phase during the esterification reaction. Thin layer chromatography analysis of (top to bottom) fatty acid ethyl esters and fatty acids. The reaction system comprised 2.82 g of fatty acid and 580 μl of ethanol added in a three-step manner to 0.33 g of immobilized lipase at 30°C for 5 hours and spun at 190 rpm.

Esterification was performed at 30°C in a 50-ml screw-cap tube containing 2.82 g of FFA distilled from soybean oil hydrolysis products and 0.33 g of immobilized lipase membrane, with the addition of 580 μl of ethanol using a three-step method. Esterification degree and acid value were determined for the overall process (Figure 5A), and each sample was developed on a TLC plate by using petroleum ether, ethyl ether and acetic acid (Figure 5B). The results indicate that the reaction velocity was fast, the degree of esterification reached 85% after 3 hours and the product was pure, that is, it consisted solely of FAEE.

### Stability of immobilized lipase catalysis system

**Figure 6:**
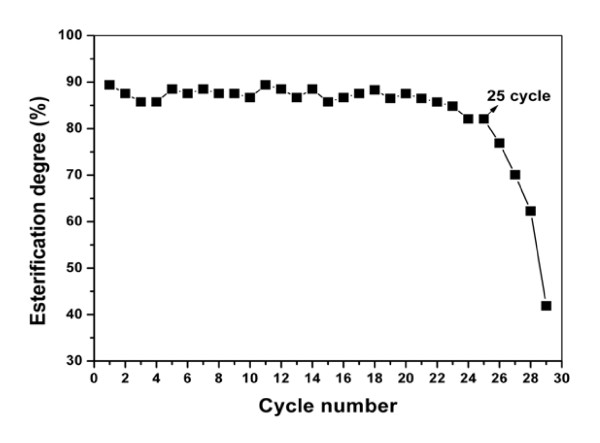
**The stability of the esterification reaction**. The reaction system comprised 2.82 g of fatty acid, 580 μl of ethanol added in a three-step manner, 9% (wt/vol) molecular sieves added 1 hour after the start of the reaction and 0.33 g of immobilized lipase at 30°C for 5 hours and spun at 190 rpm. After each batch, immobilized lipase membrane was taken out, dried and reused in a fresh reaction.

Immobilized lipase membrane prepared as described in the Methods section displayed good long-term stability. The degree of esterification was still 82% after 25 batches were run, and then it declined rapidly to 42% by the 30th batch (Figure 6).

A total of 66.6 g of FAEE were produced in 29 batches catalyzed by immobilized lipase membrane. The collected reaction solution was treated as described in the Esterification reaction section. The final product was obtained with 95% recovery, with the following composition as determined by GC analysis: 15.4 g of palmitic acid ethyl ester, 4.4 g of stearic acid ethyl ester, 18.8 g of oleic acid ethyl ester, 55.2 g of linoleic acid ethyl ester, and 6.2 g of α-linolenic acid ethyl ester per 100 g of FAEE.

## Discussion

*Y. lipolytica *lipase has been applied frequently [8,9] for the degradation of hydrocarbons and the hydrolysis of esters. It retains its activity in many organic solvents to catalyze esterification, transesterification and resolution of racemic mixtures [10-13]. These characteristics of *Y. lipolytica *lipase make it a good candidate for the catalysis of FAEE (that is, biodiesel) production.

Here the more efficient system of producing biodiesel was achieved by performing hydrolysis followed by esterification catalyzed by *Y. lipolytica *lipase. The conversion rate for the esterification reaction between fatty acids and ethanol catalyzed by *Y. lipolytica *lipase was 85% in a 3-hour reaction. In contrast, the conversion rate for esterification by *C. antarctica *lipase was only 70% in a 3-hour reaction; 5 hours were needed to reach 85% conversion. Moreover, when the method described in the Methods section was used to analyze esterification with *C. Antarctica*-immobilized lipase by a substrate of lauryl alcohol and lauric acid, esterification activity of 4,000 Ue/g was observed. A concentration of 60 Ue/g fatty acids was used to catalyze esterification in the Watanabe *et al. *report [7]. In contrast, the esterification activity of lipase was 18 Ue/g fatty acid in the present paper, with 150 Ue/g fixed fabric membrane. Consequently, a high-speed reaction and low consumption of lipase are realized when *Y. lipolytica *lipase is used.

In the esterification reaction, the *Y. lipolytica *lipase activity is inhibited by ethanol. When the molar ratio of ethanol to fatty acid was higher than 1:1, the esterification rate was reduced from 72.7% to 54.4% or less (Figure 3B). The inhibitory effect of ethanol was reduced significantly when a multistep addition method was used (Table 1). We compared the inhibition of esterification by methanol and ethanol using the same molar concentrations and the same three-step addition method. After 3 hours, the conversion rates of methanol and ethanol, as measured by acid-base titration, were 53.6% and 82.6%, respectively. This finding differs from that of Watanabe *et al. *[7], who found that the inhibitory effect of methanol on immobilized enzyme in a lipase-catalyzed esterification system was much higher than that of ethanol, which might be due to the difference in lipase source.

The esterification reaction can be promoted by controlling the water content in the mixture. The conversion rate was increased to 5% if a molecular sieve was added to remove water, and the rate was lowered by approximately 5% if 3% (vol/vol) water was retained during the reaction (Figure 4). The inhibitory effect of water on esterification could be restored following the removal of water by the molecular sieve or after drying lipase membranes finished the esterification. So, we suggest that water had no effect on enzyme activity, but affected only the esterification equilibrium. In contrast, in the *Candida sp*. lipase-catalyzed transesterification system studied by Tan *et al. *[2], a certain amount of water promoted the reaction. One possible explanation is that water is first involved in hydrolysis of triglycerides, and then the lipase catalyzes esterification of the hydrolyzed fatty acids. Further study is needed to determine whether water is really involved in the transesterification reaction.

Interestingly, unsaturated fatty acids are preferable for use with *Y. lipolytica *lipase. We found that reactions in which oleic acid was subtracted could be conducted for 91 batches in the same esterification system [14], whereas fatty acid distillation can reach only 25 batches. This preference is also reflected in the composition of fatty acids and fatty acid ethyl esters resulting from the two-step process. The fatty acid composition revealed by GC analysis was 20.0% palmitic acid, 4.9% stearic acid, 24.2% oleic acid, 47.0% linoleic acid and 3.9% linolenic acid, whereas fatty acid ethyl esters consisted of 15.4% palm ethyl ester, 4.4% stearic acid ethyl ester, 18.8% ethyl oleate, 55.2% ethyl linoleate and 6.2% ethyl linolenate. The proportions of saturated vs. unsaturated fatty acid ethyl esters were 19.8% and 80.2%, respectively, in contrast to corresponding proportions of 24.9% and 75.1% for fatty acids.

Although the two-step catalysis system has resolved the inhibition of glycerol and removed the reaction solvents, hydrolysis efficiency is low. In the present study, the major parameters affecting the hydrolysis reaction catalyzed by *Y. lipolytica *lipase were optimized and the oil/water interface was increased by the addition of an emulsifier; however, the hydrolysis degree of 91.9% was obtained within 48 hours. There is a possible reason that *Y. lipolytica *lipase is a 1,3-specific lipase and does not act on the 2 site of the ester bond of triglyceride. Only the 2 site of the ester bond was actually shifted spontaneously to either the 1 or the 3 site, and the hydrolysis reaction was continued [15]. In practice, such spontaneous shifts happen slowly in aqueous media. Thus nonspecific lipase must be prepared through the selection of other kinds of lipase or by using some other molecular biological method.

## Conclusions

In comparison to transesterification methods, the enzymatic method described here has some advantages [2]. It has a reduced inhibitory effect on lipase activity and independence from petroleum sources, since ethanol is used as a substrate. Fatty acids and ethanol are well mixed on the basis of their similar polarity, and esterification reactions can be completed within 3 hours. It leads to improved safety of production by avoiding the use of low-boiling-point organic solvents such as petroleum ether and *tert*-butanol alcohol. Conducting a two-step method does not produce glycerol during esterification, does not mask the enzyme and extends the life of the immobilized enzyme. More efficient industrial production is realized, since only one low-cost lipase is used. The acid-base titration method was used to track the course of esterification, which is convenient for inspection in an industrial setting. These advantages indicate that the two-step protocol used in this study may be applicable to an industrial process for the production of biodiesel fuel from vegetable oil, especially from high acid value waste oil.

## Methods

### Raw materials

Soybean oil was purchased from Qinhuangdao Jinhai Grain & Oil Industrial Co., Ltd. (Qinhaungdao, China). Its fatty acid composition was palmitic acid 20.03%, 4.85% stearic acid, 24.17% oleic acid, 47.03% linoleic acid and 3.92% α-linolenic acid. Olive oil was purchased from Sinopharm Chemical Reagent Co., Ltd. (Shanghai, China). Heptadecanoic acid methyl ester (chromatographically pure) was from Sigma, USA. *Yarrowia lipolytica *strain was from China Agricultural University. (The strain was deposited at the China General Microbiological Culture Collection as CGMCC 2707.) Its lipase was produced by Qinhuangdao Leading Science & Technology Co., Ltd. **Qinhuangdao, China. **Soybean powder was obtained from a local market. All other reagents were obtained commercially and were of analytical grade.

### Lipase preparation

The *Y. lipolytica *strain CGMCC 2707 was stored at -80°C in tubes containing 25% (vol/vol) glycerol solution. For the preparation of inoculum, cells were transferred to YPD medium (20 g of tryptone, 10 g of yeast extract and 20 g dextrose per liter autoclaved for 15 minutes at 121°C) two times for activation, and they were then incubated at 28°C. Activated cells were inoculated into fermentation medium, which contained 60 g of soybean powder, 90 g of soybean oil, 2.5 g of K_2_HPO_4_, 0.5 g of MgSO_4_·7H_2_O and 2 g of (NH_4_)_2_SO_4 _per liter of distilled water. Thirty-liter cultures were grown in a 50-l fermentor with agitation at 500 rpm and 1:1 vvm air flow at 28°C, and pH was adjusted to 6.5 by using 10 N KOH. The lipase produced reached 8,000 Uw/ml after 90 to 110 hours of fermentation (Uw refers to the hydrolysis activity of lipase). Lipase solution was obtained by the removal of cells by centrifugation (4,000 × *g *for 20 minutes).

Lipase in the supernatant was precipitated by the addition of three volumes of acetone. The precipitate was washed with acetone and dried at room temperature. The activity of the enzyme powder was 140,000 Uw/g.

### Immobilization of lipase on fabric membrane

Lipase was immobilized by using an established immobilization procedure [16]. Briefly, 0.1 g of fabric (approximately 9 cm^2^) was presoaked for 1 hour in 10 ml of coimmobilization solution consisting of 0.5 g of gluten, 0.2 g of lecithin, 0.2 g of polyethylene glycol 6000 and 0.1 g of magnesium chloride. Fabric membranes were dried at room temperature and used as supports for the immobilization of lipase. Membranes were added into 10 ml of enzyme solution (5,000 to 10,000 Uw/ml), stirred for 2 to 3 hours, taken out and dried at room temperature under a vacuum. The activity of immobilized lipase, determined by using an olive oil emulsion method after grinding at 0°C, was 10,000 Uw/g membrane.

### Lipase activity determination

Hydrolysis activity of lipase (Uw) was determined by using the olive oil emulsion method. One hydrolysis activity unit was defined as the amount of enzyme required to release 1 μM fatty acid per minute under assay conditions [6].

The esterification activity of lipase (Ue) was determined by using a lauric acid and lauryl alcohol reaction system. One esterification activity unit was defined as the amount of enzyme required to release 1 μM lauric lauryl ester per minute under assay conditions. The substrate was an equimolar mixture of lauric acid with lauryl alcohol at a final concentration of 0.1 mM in *n*-hexane solvent. The reaction was initiated by adding 0.01 g of lipase (pure or diluted depending on the activity of lipase), continued by incubation for 20 minutes at 40°C and stopped by the addition of 15 ml of ethanol. Enzyme activity was determined by titration of the remaining lauric acid with 100 mM sodium hydroxide. Esterification activity was calculated on the basis of the release of lauric lauryl alcohol using the following formula:

$$\text{Ue}=\left[\left(V_{0}-V_{\text{NaOH}}\right)\times 0.\text{1}/\text{2}0\right]\times \text{1},000,$$

where *V*_0 _and *V*_NaOH _are, respectively, the volumes of NaOH consumed by titration of the mixture at the beginning (0 minutes) and end (20 minutes) of the reaction.

### Hydrolysis reaction

Small-scale hydrolysis was conducted at 40°C in a 50 ml screw-cap tube containing 2 g of soybean oil, 200 Uw lipase broth and 1.2 ml of water, with agitation on an orbital shaker (180 rpm) for 28 to 48 hours. At defined intervals, 0.8 ml of the reaction mixture was removed and separated into oil and water phases by centrifugation (10,000 × *g *for 5 minutes). The oil phase was analyzed as described in the Analytical methods section.

Large-scale hydrolysis was conducted by filling a 1-l reaction vessel with 200 g of soybean oil, 20,000 Uw lipase broth, 120 ml of water and 10 g of sodium stearate and then agitating the mixture at 180 rpm at 40°C. When the degree of hydrolysis reached 90%, the reaction mixture was acidified with 3 N sulfate until the pH of the water layer was 4.5. The water layer was then removed, and the remaining oil layer was washed twice with hot water (70°C to 80°C). The oil layer was vacuum-distilled at 93 to 98 kPa, and distillates were collected at 220°C to 260°C. The resulting fatty acid fraction was used for esterification as described below.

### Esterification reaction

Fatty acid was esterified using immobilized lipase membrane in 50-ml stoppered flasks without organic solvent. The reaction was performed with 2.82 g of oleic acid or FFA and 0.33 g of lipase membrane, and 192 μl of ethanol were added every 1 hour (oleic acid:ethanol molar ratio, 1:0.3) until theoretical molar ratio was reached. The mixture was incubated with agitation at 130 rpm at 30°C. Molecular sieves (Figure 5A) were added for 1 hour to eliminate water. Immobilized lipase and fatty acid were preheated in a 30°C incubator for 30 minutes, and the reaction was started by the addition of ethanol to the system. Experiments were replicated three or more times, and the results are presented as mean values.

Adsorbed water and lipase membranes were recovered from the reaction solution by filtration, and 15% (wt/vol) NaOH solution was added according to the amount of remaining fatty acid. The solution was stirred slowly for 30 minutes and then left undisturbed so that the aqueous and organic phases could separate. The organic phase was washed twice with two volumes of water to remove unreacted ethanol and dehydrated by decompression distillation. The final product, ethyl ester (biodiesel), was obtained with 95% recovery.

### Analytical methods

#### TLC

Silica gel plates (Whatman Inc. Shaihai, China) were heated at 110°C for 1 hour prior to use. Oil phase samples obtained as described in the small-scale hydrolysis reaction section were dissolved in acetone to form a 10 mg/ml solution, and 10-μl capillary spots were subjected to TLC analysis. The spots were sprayed with a 20 volume percent solution of sulfuric acid in ethanol developed with petroleum ether/ethyl ether/acetic acid (80:30:1 ratio, volume fraction) and visualized by heating at 100°C for 30 to 50 seconds.

Gas chromatography was conducted to quantify the composition of fatty acids and FAEEs. At a predefined time, 20-μl samples were taken and centrifuged. A quantity of 5 μl of the upper phase thus obtained was dissolved in *n*-hexane and analyzed using a GC-2010 gas chromatograph (Shimadzu, Kyoto, Japan) equipped with a capillary column (HP-INNOWax columns, 30 m-0.25 mm-0.25 μm; J & W Scientific Columns, Agilent Technologies, Palo Alto, **USA**) and a flame ionizing detector. Injection was performed in split mode (1:30), with injection and detection temperatures of 260°C and 280°C, respectively. Samples (1 μl) were injected at an oven temperature of 240°C and held for 10 minutes. The carrier gas was nitrogen at a flow rate of 30 ml/min.

Hydrolysis degree was calculated as the acid value of the hydrolyzed oil sample as a percentage of the saponification value of soybean oil.

The degree of esterification was calculated as the reduction of acid value (obtained by titration of aliquots of mixture taken at the beginning and end of the reaction) as a percentage of fatty acid value.

Water content was measured by using a Karl-Fisher WS-3 trace moisture analyzer (Shandong Zibo Corson Instruments, Zibo, China) [17].

The viscosity of the oil or reaction mixture was measured using a viscometer (RVDV-II+PRO; Brookfield Engineering Laboratories, Middleboro, MA, USA).

## Competing interests

The authors declare that they have no competing interests.

## Authors' contributions

YL and JL designed and coordinated the study. YM and GW carried out the experiments. NY, YL, ZZ, XL and JC analyzed the results. YM and GW wrote the paper, and YL and JL reviewed the paper. All authors read and approved the final manuscript.
